# Somatic mutational landscape reveals mutational signatures and significantly mutated genes of cancer immunotherapeutic outcome and sex disparities

**DOI:** 10.3389/fimmu.2024.1423796

**Published:** 2024-11-01

**Authors:** Yuting Li, Qinghua Wang, Xiaopan Gao, Jinyang Zheng, Wenjing Zhang, Yanfeng Ren, Wei Shen, Wei Su, Ping Lu

**Affiliations:** ^1^ Department of Radiation Oncology, Department of Pathology, Life Science Research Center, The First Affiliated Hospital of Xinxiang Medical University, Xinxiang, Henan, China; ^2^ Department of Health Statistics, Key Laboratory of Medicine and Health of Shandong Province, School of Public Health, Shandong Second Medical University, Weifang, Shandong, China; ^3^ Department of Pulmonary and Critical Care Medicine, Sunshine Union Hospital, Weifang, Shandong, China; ^4^ Department of Oncology, The First Affiliated Hospital of Xinxiang Medical University, Xinxiang, Henan, China; ^5^ Department of Pathology, The First Affiliated Hospital of Xinxiang Medical University, Xinxiang, Henan, China

**Keywords:** cancer immunotherapy, mutational signatures, molecular subtypes, significantly mutated genes, molecular indicators

## Abstract

**Background:**

Currently developed molecular markers can predict the effectiveness of cancer immunotherapy and screen beneficiaries to some extent, but they are not stable enough. Therefore, there is an urgent need for discovering novel biomarkers. At the same time, sex factor plays a vital role in the response to immunotherapy, so it is particularly important to identify sex-related molecular indicators.

**Methods:**

We integrated a pan-cancer cohort consisting of 2348 cancer patients who received immune checkpoint inhibitors and targeted sequencing. Using somatic mutation profiles, we identified mutational signatures, molecular subtypes, and frequently mutated genes, and analyzed their relationships with immunotherapeutic outcomes. We also explored sex disparities of determined biomarkers in response to treatments.

**Results:**

We found that male patients exhibited better immunotherapy outcomes and higher tumor mutational burden. A total of seven mutational signatures were identified, among which signatures 1 and 3 were associated with worse immunotherapy outcomes, while signatures 2 and 6 correlated with better outcomes. Gender-based analysis revealed that mutational signature 1 continued to show a worse immunotherapy outcome in female patients, whereas signature 6 demonstrated a better outcome in male patients. Based on mutational activities, we identified four potential molecular subtypes with gender differences and relevance to treatment outcomes. PI3K-AKT, RAS signaling pathways, and 68 significantly mutated genes were identified to be associated with immunotherapy outcomes, with nine genes (i.e., ATM, ATRX, DOT1L, EP300, EPHB1, NOTCH1, PBRM1, RBM10, and SETD2) exhibiting gender differences. Finally, we discovered co-mutated gene pairs and TP53 p.R282W mutations related to treatment outcomes, highlighting their gender-specific differences.

**Conclusion:**

This study identified several molecular biomarkers related to cancer immunotherapy outcomes in terms of mutational signatures, molecular subtypes, and mutated genes, and explored their gender-relatedness in order to provide clues and basis for clinical treatment efficacy evaluation and patient selection.

## Introduction

Cancer immunotherapy has revolutionized the treatment landscape, and among its most promising approaches is the use of immune checkpoint inhibitors (ICIs) (1). These drugs target specific molecules, known as checkpoints (e.g., PD-1, PD-L1, or CTLA-4), that are involved in regulating the immune system’s response to cancer cells. The use of ICIs has shown remarkable success in treating a variety of cancers (2), including melanoma, lung cancer, and kidney cancer. However, these drugs are not effective in all patients, only a small percentage of patients could benefit from it (3). To optimize the use of immune checkpoint inhibitors, oncologists often consider a patient’s tumor characteristics, such as PD-L1 expression levels and tumor mutation burden (TMB). However, PD-L1 expression can vary greatly among different tumors, which can limit the effectiveness of PD-L1 inhibitors (4). The accuracy of TMB assessment can be influenced by factors such as sample quality, detection methods, and bioinformatic analysis (5). Therefore, novel and stable molecular biomarkers are urgently needed.

Mutational signature refers to the pattern of genetic mutations that occur in a cell or tissue, which can be caused by various endogenous and exogenous factors (6). These signatures are unique to each individual and can be used to identify the underlying causes of mutations, such as exposure to carcinogens, DNA repair defects, or inherited genetic diseases. Mutational signatures can be utilized to predict the response of patients to immunotherapy (7). Smoking and APOBEC-related mutational signatures are associated with a favorable prognosis and response to immunotherapy (8, 9), while defective DNA repair signature may indicate resistance to treatment (3, 5). Mutational signatures provide a powerful tool for understanding the complex interactions between genetics, immunology, and cancer. By analyzing these signatures, researchers can gain insights into the underlying causes of mutations, the immune response to them, and the potential for immunotherapy to treat associated cancers.

The utility of individual gene mutations in assessing tumor immunogenicity and treatment response has been demonstrated. Li et al. showed that MUC16 mutations are associated with better immunogenicity and prognosis in gastric cancer (10). Using integrated immunotherapy cohorts, Zhang et al. identified FAT1 mutations as predictors of improved ICI response rates and clinical outcomes in both melanoma and non-small cell lung cancer (NSCLC) (11). Additionally, POLE, PBRM1, TTN, B2M, and JAK1/2 mutations have also been shown to be associated with immunotherapy response or resistance (5). These findings suggest that mutations in specific genes may play a crucial role in determining the efficacy of immunotherapy.

Recent studies have highlighted the significant gender differences in immunotherapy response, which can be attributed to various factors such as hormonal differences and immune system differences (12). Compared to males, anti-PD-1/L1 therapy has been shown to improve overall survival and response rates among female patients with NSCLC (12). Conversely, in colorectal cancer, male individuals have significantly prolonged survival than females (12). For melanoma, six out of seven clinical trials have demonstrated higher overall survival rates with ICI treatment among male patients compared to female patients (12). Klein et al. discussed how males often have better outcomes with tumor necrosis factor (TNF) inhibitors in autoimmune conditions, whereas females may experience more adverse effects and lower remission rates (13). This study highlighted the broader implications of sex differences in immune responses and treatment adherence. Ma et al. explored sex differences in the efficacy of ICIs in cancer treatment, showing that males often responded better, while females may benefit more from combination therapies (14). They also examined how hormonal influences contributed to these differences. A recent study reviewed the impact of sex hormones, immune system differences, and genetic factors on cancer immunotherapy outcomes. It emphasized the need for sex disparity analyses in clinical trials to improve personalized treatment strategies (15).

In this study, we integrated somatic mutation profiles and clinical immunotherapy information from a total of 2384 patients with nine distinct cancer types. We explored molecular markers associated with immunotherapy outcomes from three perspectives: mutational signatures, molecular subtypes, and mutated genes. Additionally, we analyzed their gender disparities to aid in the achievement of personalized treatment.

## Methods

### Collection of genomic data and clinical information

**Figure 1:**
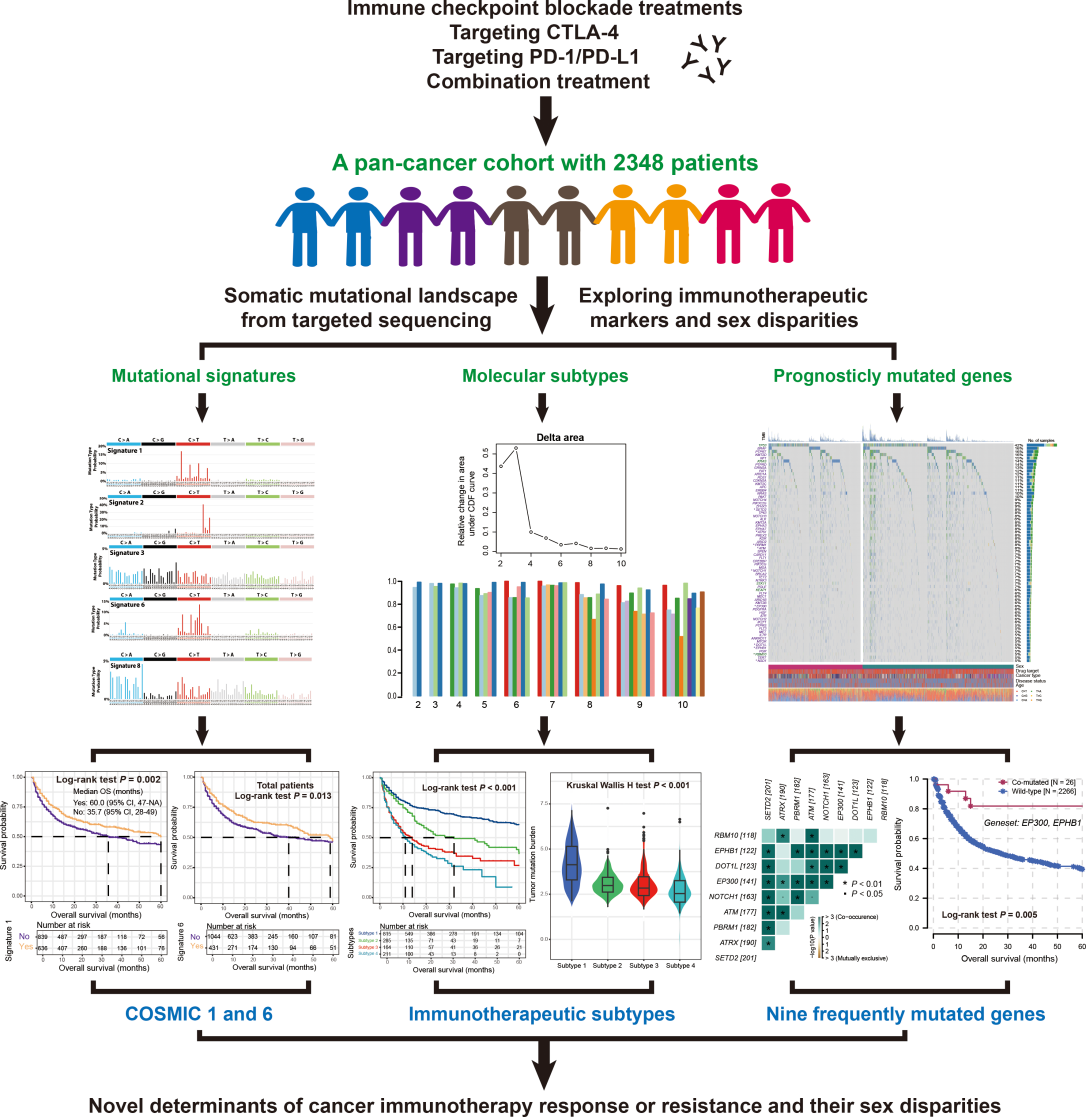
Research design of this study to discover novel mutational molecular determinants of cancer immunotherapy outcome and sex disparities.

A total of 2348 cancer patients from Memorial Sloan Kettering Cancer Center (MSKCC) were collected (16–19), who had undergone immune checkpoint inhibitor (ICI) treatments (i.e., anti-PD-1/L1, anti-CTLA-4, or combination) and Integrated Mutational Profiling of Actionable Cancer Targets (MSK-IMPACT) targeted sequencing. The final patients included nine tumor types (i.e., SKCM [n = 800], NSCLC [n = 571], BG [n = 115], BLCA [n = 212], BRCA [n = 43], CRC [n = 110], EGC [n = 123], HNSC [n = 138], and RCC [n = 150]), as well as 86 patients with unknown types. Totaling 37289 somatic mutations were obtained for relevant analysis. Detailed clinicopathologic and immunotherapeutic information across all cancer patients were illustrated in **Supplementary Table S1**. Detailed study design was shown in **Figure 1**.

### Determination of mutational signatures operative in the genome

The recently proposed Signature Multivariate Analysis (SigMA) method (20) by Gulhan et al. was utilized for the extraction of mutational signatures. Unlike previous tools that rely on whole-exome or whole-genome sequencing data, SigMA can detect mutational signatures using a relatively low number of mutations, based on likelihood methods and machine learning. This makes it particularly suitable for targeted sequencing mutation data. All identified mutational signatures were compared with the 30 annotated signatures in the COSMIC database (version 2) (21) to obtain the final mutational activity. Finally, the identified mutational signatures were converted into binary variables (yes or no) for related analyses, according to a recent study (22): a mutational signature is considered to be present in a sample if it contributes to more than 100 mutations or exceeds 25% of the mutational activity.

### Molecular subtyping based on consensus clustering analysis

We identified molecular subtypes using the mutational activity data corresponding to the extracted mutational signatures. Consensus clustering analysis was performed using the partition around medoids (PAM) method with Euclidean distance and performed 500 bootstraps each comprising 80% of samples based on the R ConsensusClusterPlus package (23). To obtain a more accurate number of clusters, we set the clustering range from 2 to 10, and ultimately determined the final subtype count based on the consistency clustering coefficient and consistency matrix.

### Prognostic mutated genes and related signaling pathways

Univariate Cox regression analysis was employed to screen mutated genes related to immunotherapy prognosis. For genes with *P* values less than 0.05, we performed pathway enrichment analysis based on the KEGG and GO databases using functions from the clusterProfiler package (24). This allowed us to identify enriched pathways for prognosis-related genes and to identify signaling pathways related to immunotherapy outcomes. Subsequently, we focused on prognosis-related genes with mutation frequencies greater than 0.05 and analyzed their prognostic relevance in different genders. The specific mutation patterns of these genes were visualized using a waterfall plot from the maftools package (25).

### Gene pairs of co-mutations or mutual exclusion

Co-mutations refer to the phenomenon of multiple mutations occurring in distinct genes within the same genome, while mutually mutational exclusion refers to a low degree of overlap in mutations between genes. Studies have shown that co-mutations are one of the core determinants of cancer and are associated with prognosis and drug sensitivity (26). In this study, co-mutation genes and mutually mutational exclusion genes were identified using the maftools package (25), and their occurrence in different genders was analyzed. Furthermore, gender-related co-mutation gene pairs were identified.

### Statistical analyses

The analyses and graphical representations in this study were primarily conducted using relevant packages in the R software. The significance of differences in TMB between different genders was analyzed using the Wilcoxon rank-sum test, and box plots for dichotomous variables were generated. Differences in TMB among distinct molecular subtypes were assessed using the Kruskal-Wallis H test, and box plots for multinomial variables were plotted. Kaplan-Meier methodology was employed to generate survival curves, and the significance of differences between curves was tested using the log-rank method. A multivariate Cox regression model including clinical factors such as age, disease status, tumor type, and drug target was utilized to adjust for confounding variables, and this analysis was implemented using the forestmodel package.

## Results

### Male patients showed higher TMB and better immunotherapy outcome compared to females

**Figure 2:**
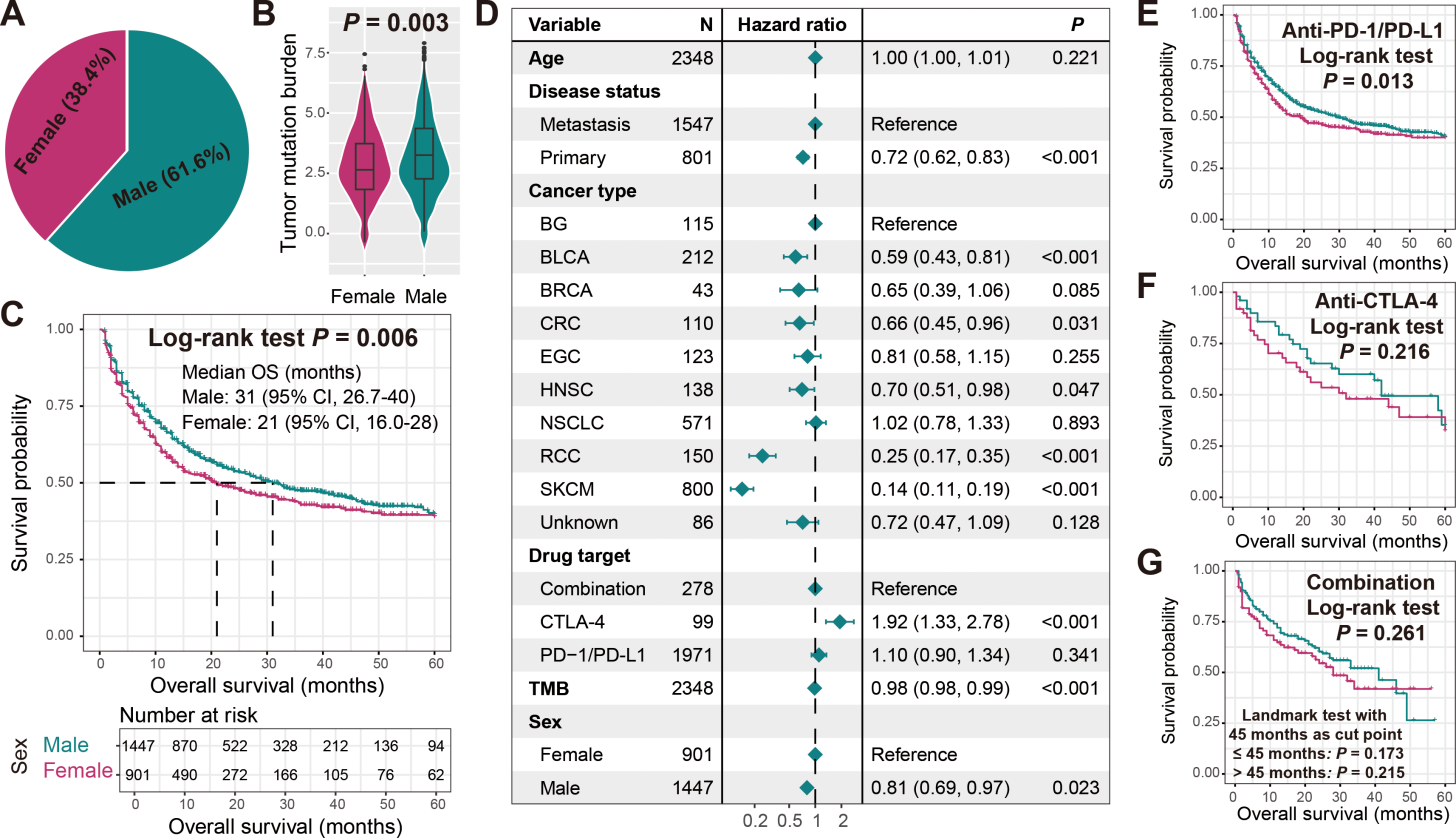
Association of sex with TMB and immunotherapy outcome. **(A)** Sex percentage of all cancer samples included in this study. **(B)** Boxplot illustration of the connection of sex with TMB. **(C)** Immunotherapeutic survival curves stratified by male and female patients. **(D)** Multivariate Cox regression model of sex with age, disease status, cancer type, drug target, and TMB taken into account. Associations of sex with ICI treatment outcome in cancer patients treated with **(E)** anti-PD-1/L1 agents, **(F)** anti-CTLA-4 agents, and **(G)** combined agents.

The median age of all 2348 included cancer patients was 63, with 1547 (65.9%) metastatic patients and 801 (34.1%) primary patients. A total of 1971 (83.9%) patients received anti-PD-1/L1 treatment, 99 (4.2%) patients received anti-CTLA-4 treatment, and 278 (11.9%) patients received combined treatment. Male patients accounted for 61.6% and female patients accounted for 38.4% (**Figure 2A**).

We observed that male patients exhibited significantly higher TMB compared to female patients (Wilcoxon rank-sum test *P* = 0.003; **Figure 2B**). Further Kaplan-Meier survival analysis revealed that male patients harbored significantly improved immunotherapy ICI outcomes (Log-rank test *P* = 0.006; **Figure 2C**). A multivariate Cox regression model including age, disease status, tumor type, drug target, and TMB still yielded consistent results (HR: 0.81, 95% CI: 0.69-0.97, *P* = 0.023; **Figure 2D**). Subsequently, we also explored gender-specific prognostic differences among patients receiving different treatment modalities. Among patients receiving anti-PD-1/L1 therapy, males still had a preferable prognosis (Log-rank test *P* = 0.013; **Figure 2E**), while in patients receiving anti-CTLA-4 and combination therapy, although not statistically significant, male patients exhibited a relatively better prognostic trend (**Figures 2F, G**). The correlations of other clinical factors such as age, disease status, tumor type, treatment type, and TMB with treatment outcomes were presented in **Supplementary Figures S1A–E**.

### Mutational signatures 1, 2, 3, and 6 associated with immunotherapeutic outcome and their sex disparities

A total of 37289 somatic mutations were included in this study, primarily missense mutation SNPs dominated by C > T mutations, with a median mutation count of 7 across all samples (**Supplementary Figure S2**). Detailed SNP transitions and transversions, as well as the types of base substitutions in all samples, were presented in **Supplementary Figure S3**. We also compared the TMB of the integrated cohort in this study with 33 tumor types in the TCGA database and found it to be at a relatively low level (**Supplementary Figure S4**). This may be due to the fact that the samples included in this study were experienced targeted sequencing with a small sequencing scope.

**Figure 3:**
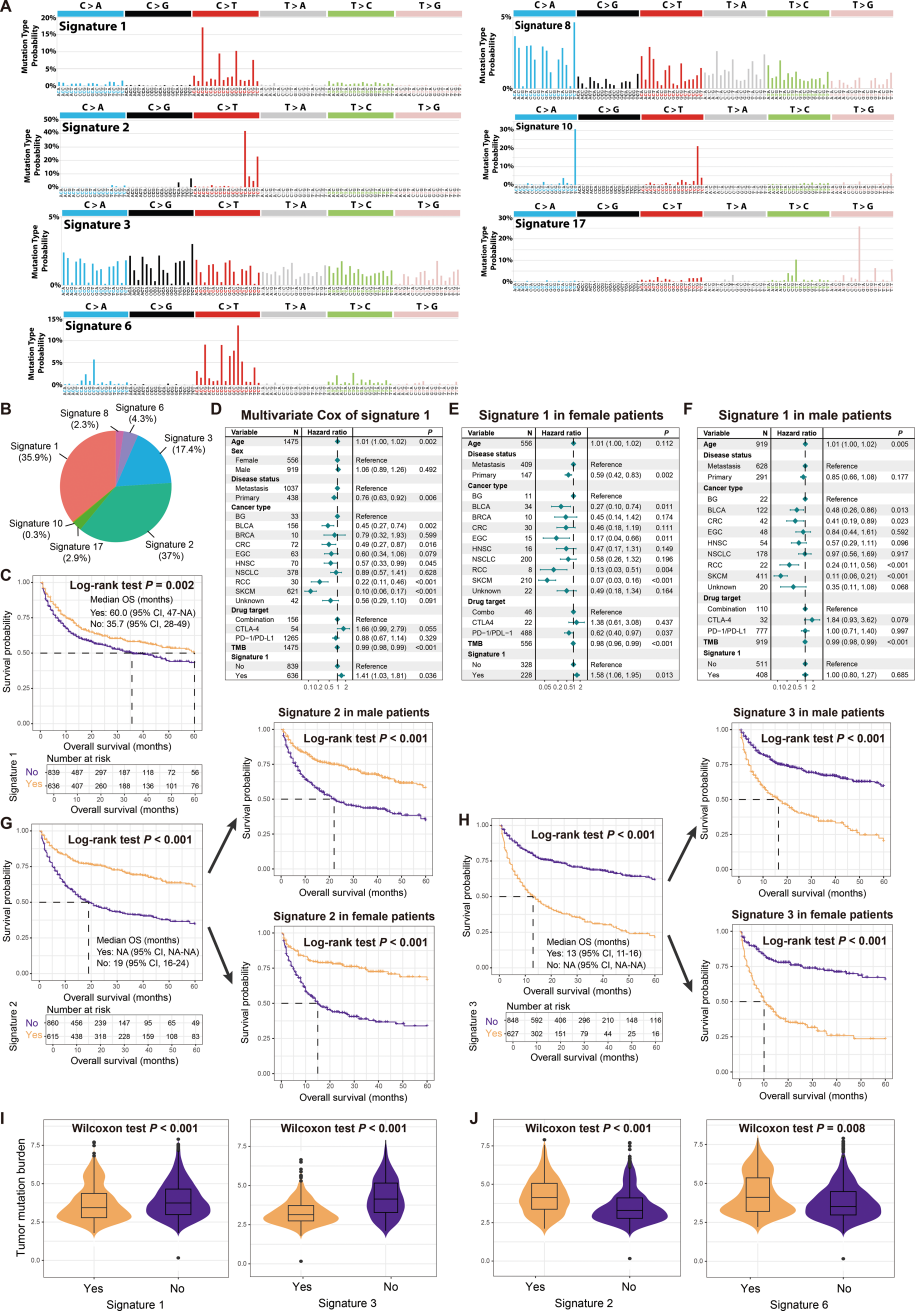
Determined mutational signatures and their associations with immunotherapy outcome and sex differences. **(A)** Detailed mutational patterns of determined seven mutational signatures (derived from the COSMIC database). **(B)** Distribution of main mutational signatures across all cancer patients. **(C)** Survival analysis of patients with and without signature 1. **(D)** Multivariate Cox regression model of signature 1 with several confounding factors taken into account. Multivariate Cox regression model of signature 1 in **(E)** female patients and **(F)** male patients. **(G)** Survival analysis of patients with and without signature 2 in total, male, and female patients. **(H)** Survival analysis of patients with and without signature 3 in total, male, and female patients. **(I)** Associations of signatures 1 and 3 with TMB. **(J)** Associations of signatures 2 and 6 with TMB.

Seven mutational signatures (i.e., signatures 1, 2, 3, 6, 8, 10, 17) were extracted based on the SigMA algorithm (**Supplementary Table S2**), and their specific mutation patterns were illustrated in **Figure 3A**. Comparison with COSMIC information revealed that signature 1 is associated with diagnostic age, signature 2 with APOBEC enzyme activity, signatures 3 and 6 with DNA damage repair deficiency, signatures 8 and 17 with unknown etiology, and signature 10 mainly due to POLE mutations. The distribution of major mutational signatures in all samples was presented in **Figure 3B**.

Kaplan-Meier survival analyses indicated that patients carrying signature 1 had a favorable prognosis for immunotherapy (**Figure 3C**). However, a multivariate Cox regression model that included multiple clinical confounding factors revealed that signature 1 was associated with an inferior prognosis (HR: 1.41, 95% CI: 1.03-1.81, *P* = 0.036; **Figure 3D**), which was also observed in female patients (HR: 1.58, 95% CI: 1.06-1.95, *P* = 0.013; **Figure 3E**) but not in male patients (HR: 1.00, 95% CI: 0.80-1.27, *P* = 0.685; **Figure 3F**). In total, male and female patients, both univariate and multivariate adjusted analyses demonstrated that the presence of signature 2 was associated with a better treatment outcome (Log-rank test all *P* < 0.001, **Figure 3G**; HR: 0.73, 95% CI: 0.31-0.99, *P* = 0.013; **Supplementary Figure S5A**), while signature 3 was associated with a poorer outcome (Log-rank test all *P* < 0.001, **Figure 3H**; HR: 1.22, 95% CI: 0.99-1.50, *P* = 0.055; **Supplementary Figure S5B**). Signature 6 was observed to be linked with a favorable treatment outcome (**Supplementary Figures S5C, D**), and this connection was confirmed in male patients (**Supplementary Figure S5E**), but not significant in female patients (**Supplementary Figure S5F**). Signatures 8, 10, and 17 did not show significant associations with treatment outcome (**Supplementary Figures S5G–I**). Furthermore, we noticed that signatures 1 and 3, which exhibited treatment resistance, harbored significantly reduced TMB (Wilcoxon rank-sum test both *P* < 0.001; **Figure 3I**), while signatures 2 and 6, which exhibited a better outcome, were associated with elevated TMB (Wilcoxon rank-sum test both *P* < 0.01; **Figure 3J**).

### Four molecular subtypes with distinct treatment outcomes and sex differences

**Figure 4:**
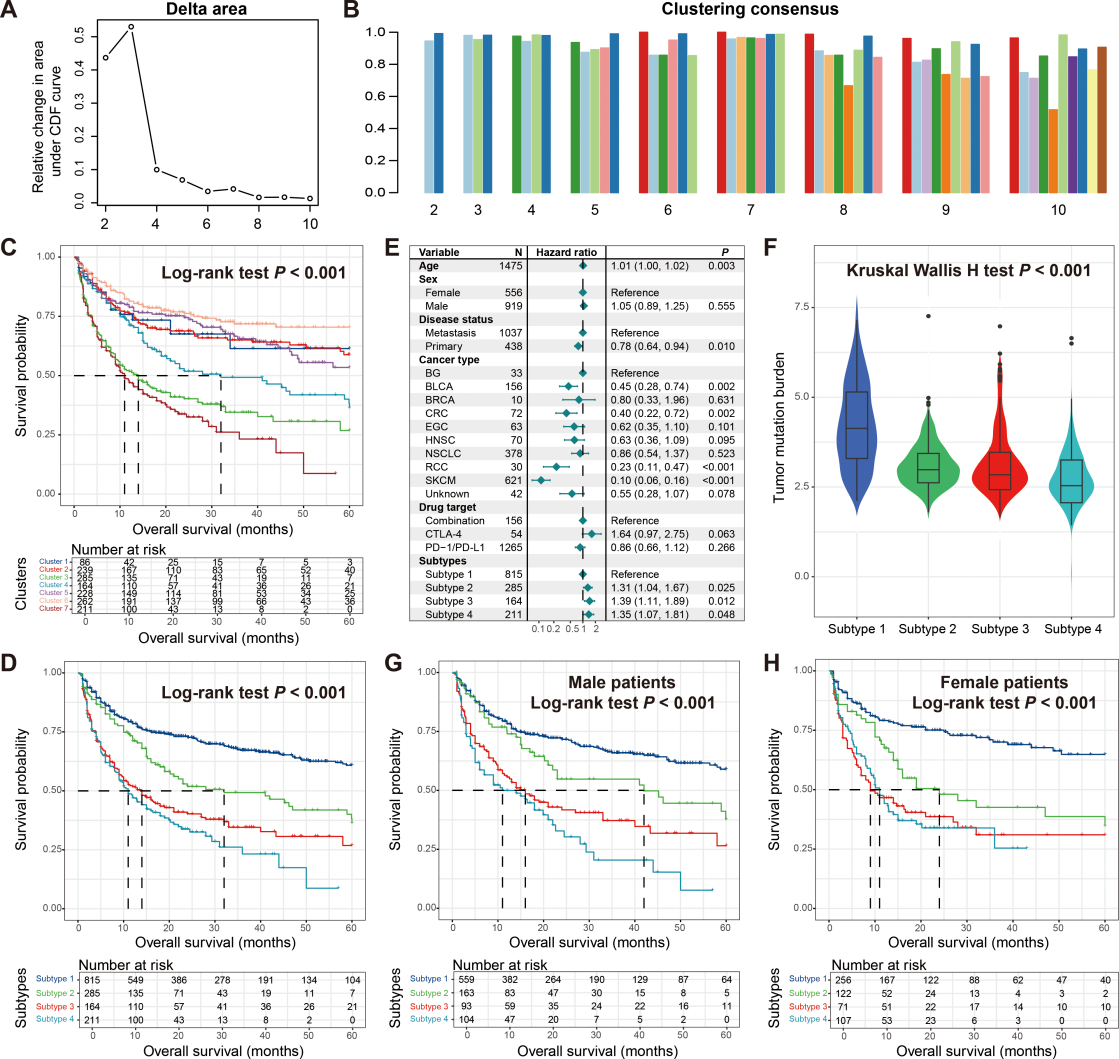
Molecular subtypes derived from consensus clustering analysis based on mutational activities. **(A)** Exhibition of clustering delta area plot. **(B)** Clustering consensus between distinct clusters with the clustering number setting from 2 to 10. **(C)** Kaplan-Meier survival curves of identified seven potential clusters. **(D)** Kaplan-Meier survival curves of determined four molecular subtypes. **(E)** Multivariate Cox regression model of four molecular subtypes with clinical confounders taken into consideration. **(F)** Distinct TMB distribution between four molecular subtypes. Kaplan-Meier survival curves of determined four molecular subtypes in **(G)** male patients and **(H)** female patients.

Based on the mutation activity data extracted from the mutational signatures, we identified sub-populations that may exist in tumor patients using consensus clustering. In this process, we set the number of clusters from 2 to 10 to screen for more reliable subtype results. The clustering trace plot was shown in **Supplementary Figure S6A**. From the clustering results, we observed that when the number of clusters was 3, the straight line dropped the fastest (**Figure 4A**), and corresponding information could also be obtained from the consensus map (**Figure 4B**, **Supplementary Figure S6B**). Survival analysis showed that these three clusters harbored significantly distinct immunotherapy outcomes (Log-rank test *P* < 0.001; **Supplementary Figure S6C**), which was also confirmed by the multivariate Cox regression correction model (**Supplementary Figure S6D**).

After further observation, we found that when the number of clusters was 7, it also exhibited good consistency (**Figure 4B**, **Supplementary Figure S6E**). From the perspective of precision medicine, we conducted further exploration. The analysis indicated that the seven identified clusters presented different prognoses, and the four with the best outcomes may be the same subtype, so we combined them into one category (**Figure 4C**). Subsequent survival analysis showed that the four molecular subtypes finally identified exhibited significantly diverse treatment prognoses (Log-rank test *P* < 0.001; **Figure 4D**), and the corrected analysis incorporating multiple confounding factors still yielded consistent results (**Figure 4E**). We noticed that subtype 1 with the best prognosis also carried the highest TMB, while other subtypes also had TMB corresponding to their outcomes (Kruskal Wallis H test *P* < 0.001; **Figure 4F**). Finally, we analyzed the differences in prognosis among these four subtypes in different genders. Male patients showed a consistent trend with the overall patient population (**Figure 4G**), while in female patients, the outcomes of the two worst-prognosis subtypes were basically the same (**Figure 4H**), indicating the gender inconsistency.

### Sex-related nine significantly prognostically mutated genes and co-mutated gene pairs

**Figure 5:**
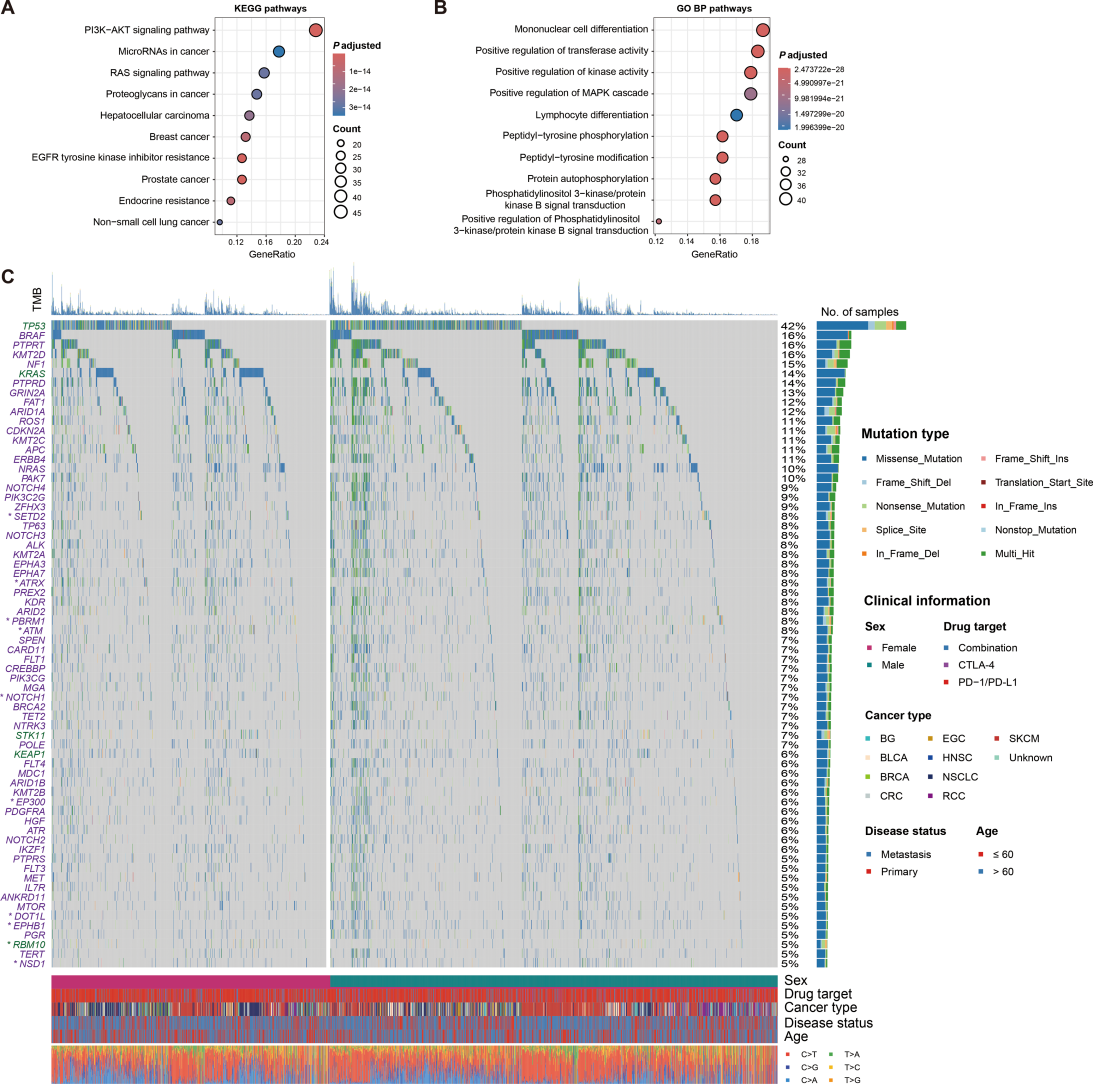
Signaling pathways and significantly mutated genes associated with treatment outcomes. **(A)** Pathway enrichment analysis of significantly prognostically mutated genes based on KEGG database. **(B)** Biological process enrichment analysis of significantly prognostically mutated genes based on GO BP database. **(C)** Waterfall plot illustration of detailed mutational patterns of immunotherapy outcome-related genes with mutation frequency greater than 0.05 and their sex differences. Genes with green were linked with inferior treatment outcomes, while with purple were linked with superior outcomes. Genes with asterisks indicated that they exhibited sex-related prognosis differences.

We performed univariate Cox regression analysis on all genes included in this study, comparing mutated versus wild-type patients, and identified 237 genes whose mutations were associated with the prognosis of immunotherapy (all *P* < 0.05; **Supplementary Table S3**). Further analysis, leveraging the KEGG and GO BP databases as background annotations, revealed significant enrichment of multiple tumor-related pathways, including the PI3K-AKT and RAS circuits (**Figure 5A**), as well as biological processes such as kinase and transferase regulation (**Figure 5B**). The results of GO CC and MF pathway enrichment were presented in **Supplementary Figures S7A, B**. The above results indicate possible signaling pathways related to the effectiveness of immunotherapy.

**Figure 6:**
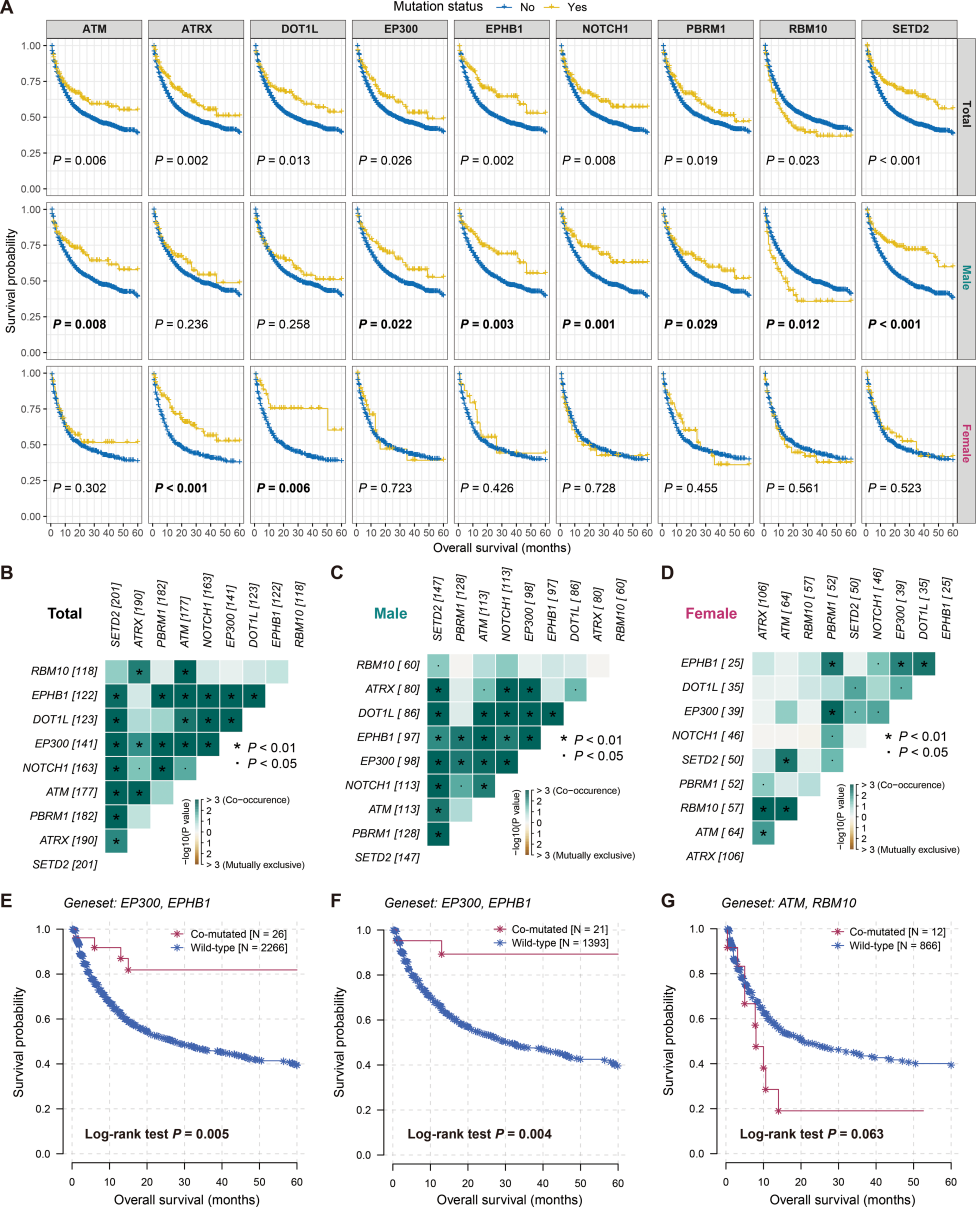
Sex-biased prognostic mutated genes and co-mutated gene pairs involved in therapeutic prognosis. **(A)** Survival analyses of nine sex-biased prognostic genes in total, male and female patients. Co-occurrence or mutually exclusion of nine sex-biased gene mutations in **(B)** total, **(C)** male, and **(D)** female patients. Kaplan-Meier survival curves of **(E)** EP300-EPHB1 co-mutated gene pair in total patients, **(F)** EP300-EPHB1 co-mutated gene pair in male patients, and **(G)** ATM-RBM10 co-mutated gene pair in female patients.

We then determined 68 prognostic genes with a mutation frequency greater than 5%, of which three genes (i.e., TP53, KRAS, and RBM10) were associated with inferior treatment outcomes, while the rest were associated with better outcomes (**Figure 5C**). Differences in mutation patterns between genders for these genes were shown with a waterfall plot. Further exploration of gender-specific effects revealed that nine of these genes (i.e., ATM, ATRX, DOT1L, EP300, EPHB1, NOTCH1, PBRM1, RBM10, and SETD2) exhibited gender-specific prognostic outcomes (**Figure 6A**). Specifically, ATM, EP300, EPHB1, NOTCH1, PBRM1, RBM10, and SETD2 mutations were associated with prognosis in male patients, while ATRX and DOT1L mutations were associated with prognosis in female patients. Correlation analyses of the above nine mutated genes in total patients, male patients, and female patients were respectively performed (**Supplementary Figures S8A–C**).

We also identified potential gene pairs that may co-mutate or mutually exclude each other among the 68 prognostic genes across all patients (**Supplementary Figure S9**, **Supplementary Table S4**). Additionally, we analyzed the potential gene pairs among the nine gender-related genes in the overall patients (**Figure 6B**), as well as in male (**Figure 6C**) and female patients (**Figure 6D**) separately. The results indicated that the co-mutation of EP300 and EPHB1 was the most significant in both the overall and male patients (both adjusted *P* < 0.001; **Supplementary Tables S5**, **S6**), and patients with this co-mutation exhibited improved immunotherapy outcomes (Log-rank test both *P* < 0.01; **Figures 6E, F**). In contrast, the most significant gene pair in female patients was ATM and RBM10 (adjusted *P* = 0.003; **Supplementary Table S7**), and patients carrying both gene mutations harbored poorer treatment prognoses (Log-rank test *P* = 0.063; **Figure 6G**).

### TP53 p.R282W mutation connected with inferior ICI outcome in female patients

**Figure 7:**
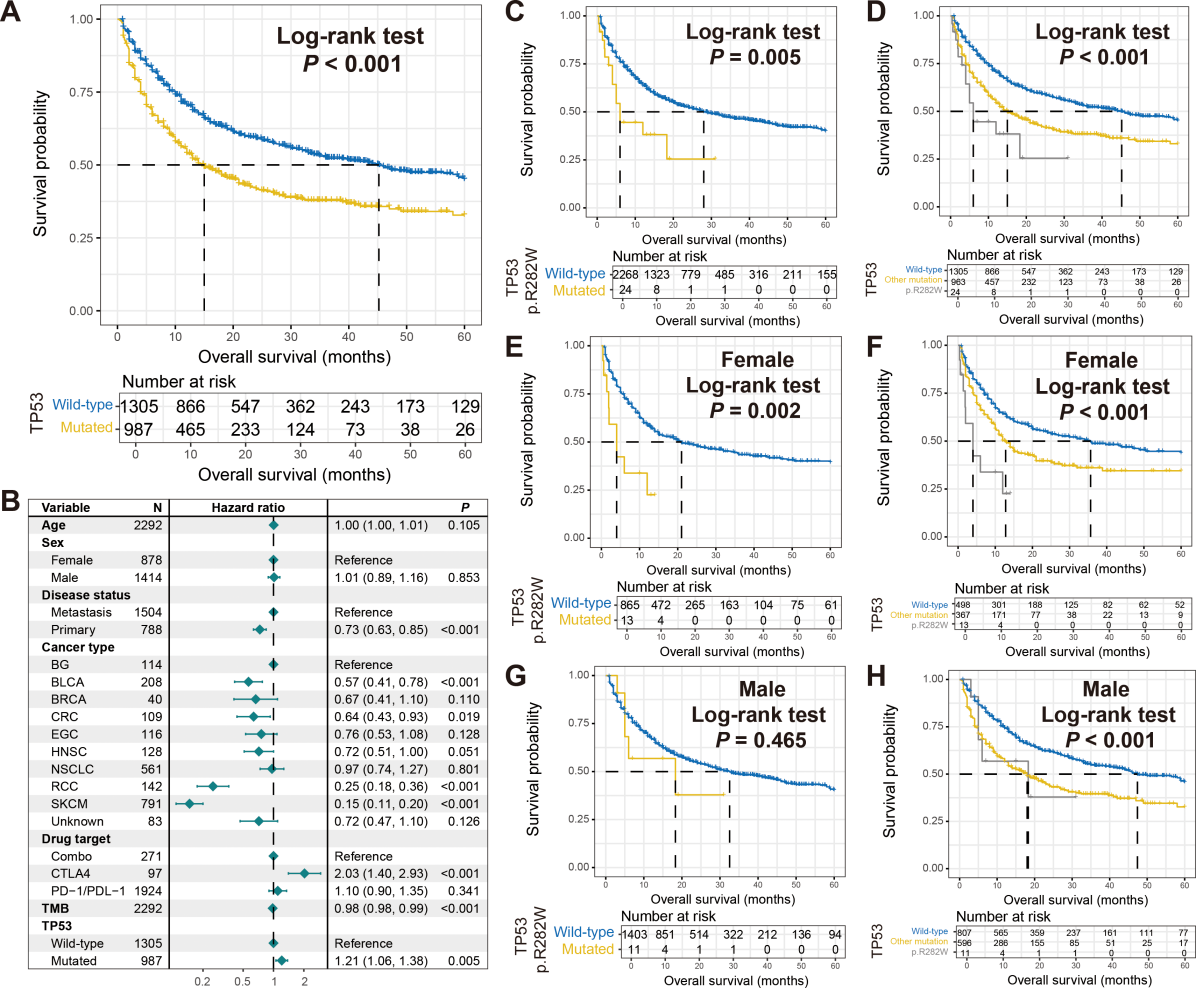
Association of TP53 p.R282W mutation with immunotherapy outcome and sex differences. **(A)** Survival analysis of patients with and without TP53 mutations. **(B)** Multivariate Cox regression model of TP53 mutations with multiple confounders adjusted. Kaplan-Meier survival curves stratified by patients with TP53 p.R282W mutations and others in **(C)** total, **(E)** females, and **(G)** males. Kaplan-Meier survival curves stratified by patients with TP53 p.R282W mutations, with other type mutations, and wild-type patients in **(D)** total, **(F)** females, and **(H)** males.

In all tumor samples included in this study, TP53 had the highest mutation rate of 42%, and its mutation was associated with poorer immune therapy outcome (Log-rank test *P* < 0.001; **Figure 7A**). This correlation was also observed in a multivariate adjusted model (HR: 1.21, 95% CI: 1.06-1.38, *P* = 0.005; **Figure 7B**). No gender-specific prognostic differences were observed for TP53 mutations (**Supplementary Figures S10A, B**). Given the need for more precise subtypes in personalized treatment, we explored distinct mutation types of TP53. The results revealed that patients with TP53 p.R282W mutation harbored significantly poorer prognosis compared to other patients (Log-rank test *P* = 0.005; **Figure 7C**), and this mutation also had worse survival compared to other TP53 mutation types (Log-rank test *P* < 0.001; **Figure 7D**). The relationship between TP53 p.R282W mutation and inferior tumor immunotherapy outcomes was further validated in female patients (**Figures 7E, F**), while male patients did not show significant associations (**Figures 7G, H**).

## Discussion

Currently, there are deficiencies in the molecular biomarkers used to evaluate the effectiveness of tumor immunotherapy, and there is an urgent need for new markers to be applied in clinical practice. This study focused on the somatic mutational landscape and sex disparities, and identified several potential molecular indicators for assessing the outcome of immunotherapy based on an integrated pan-cancer cohort, aiming to provide evidence for related clinical treatments.

Gender is a crucial factor in evaluating the effectiveness of immunotherapy, and the degree of benefit differs depending on the type of tumor. For instance, in NSCLC, female patients tended to benefit more from anti-PD-1 treatment, while male patients with colorectal cancer and melanoma experienced significantly prolonged survival (12). A recent pan-cancer meta-analysis suggested no significant difference in treatment outcomes between male and female patients (27). However, consistent with our findings, Conforti et al. discovered that male patients tended to benefit more from immunotherapy compared to female patients (28). Large-scale, prospective clinical trials are needed to further clarify the role of gender. Additionally, studies have highlighted gender differences in the utilization of molecular markers to assess the efficacy of immunotherapy (27, 29, 30).

Mutational signatures have been demonstrated to be closely associated with immunotherapy response. For instance, lung cancer patients carrying signature 4 (smoking-related signature) tended to benefit from ICI treatment (8). Patients with melanoma carrying ultraviolet light exposure mutational signature 7 exhibited elevated response rates to ICI treatment (31). The presence of APOBEC signature (signature 2) was associated with better response to anti-PD-1 treatment (9, 32), which was corroborated in our study. Furthermore, we identified age-related signature 1, homologous recombination repair deficiency-related signature 3 as associated with worse treatment outcomes, while DNA mismatch repair deficiency-related signature 6 showed better outcomes. Notably, the association between signature 1 and treatment outcomes exhibited a male bias, while signature 6 exhibited a female bias. These findings provide potential molecular determinants for tumor immunotherapy and also inform gender-based treatment strategies.

In cancer immunotherapy, molecular subtypes play a crucial role in determining the response of a patient to treatment (33). Different subtypes of tumors may have distinct immune profiles, meaning that they express different antigens and immune markers that can be targeted by immunotherapy agents. Currently, the identification of tumor molecular subtypes is primarily based on characteristic transcriptome data, such as immune-related and death pattern-related, with less focus on mutation information. Recently, the identification of molecular subtypes based on the activity of mutational signatures has been continuously reported (3, 5, 29), providing more options for the assessment of immunotherapy efficacy. In this study, by using detected seven mutational signature activities, we determined four cancer subtypes linked with diverse immunotherapeutic outcome and explored their sex differences, which provides clues for the clinical practice.

Although TMB has demonstrated some utility in predicting the efficacy of immunotherapy, its widespread application is limited by various factors such as sequencing costs, tumor types, and detection methods (34). Consequently, recent reports have focused on the use of single-gene mutations to assess tumor TMB (35, 36). Multiple studies have indicated that most individual gene mutations are associated with higher TMB levels (37). Additionally, single-gene mutations can also predict the effectiveness of immunotherapy (37). In our study, we identified several mutated genes linked with ICI outcomes and found that they were significantly enriched in pathways such as PI3K-AKT and RAS, which are deeply involved in immune regulation (38, 39), suggesting the importance of these pathways for treatment outcomes. Furthermore, we discovered nine gender-related prognostic mutated genes and identified potential gene pairs that may undergo co-mutation or mutual exclusion. Finally, the TP53 p.R282W mutation was observed to be connected with inferior outcomes and exhibit sex disparities, providing clues for individualized tumor treatment.

In this study, a total of nine mutated genes were identified to exhibit gender differences in immunotherapy outcomes. By integrating omics data and clinical treatment information from 5172 NSCLC patients, Ricciuti et al. found that ATM mutations occurred more frequently in female patients (40). A recent study indicated that female gastric cancer patients with ATRX mutations were more likely to benefit from ICI treatment (41). Holowatyj et al. conducted a gender difference analysis based on somatic mutation data from early-onset colorectal cancer patients and discovered that male patients typically had a higher mutation rate of EP300 (42). The above findings pointed to the gender differences in ATM, ATRX, and EP300 mutations across different scenarios, further providing evidence for the gender differences observed in immunotherapy outcomes in this study. However, no literature has reported clinical differences in DOT1L, EPHB1, NOTCH1, PBRM1, RBM10, and SETD2 mutations between genders, suggesting that they may be novel gender-related molecular markers. Prospective clinical trials and functional experiments are needed to further validate these findings.

Shi et al. identified mutational molecular biomarkers associated with treatment outcomes by integrating whole-exome sequencing (WES) somatic mutation data and clinical immunotherapy information from melanoma (29). Specifically, CFH mutations exhibited better ICI prognosis and higher response rates in male patients, while smoking-related mutational signature 4 correlated with poorer treatment outcomes in males. In female patients, an immune molecular subtype predictive of worse outcomes was identified. Compared to the study by Shi et al., the present study offered several advantages. Firstly, it integrated mutation data and immunotherapy information from patients with nine types of tumors, enabling the revelation of treatment-related molecular markers at a pan-cancer level with broader application. Secondly, the larger sample size allows for more statistically reliable results. Thirdly, this study identified co-mutated gene pairs associated with immunotherapy response, providing novel insights for clinical practice.

There are generally two methods to obtain tumor somatic mutation data: WES and targeted sequencing. For WES mutation data, the commonly used mutational signature extraction methods are SignatureAnalyzer proposed by Kim et al. (43) and the built-in method in the R maftools package (25). Both of these methods extract mutational signatures based on the principle of Bayesian non-negative matrix factorization. The mutation data included in this study was obtained through MSK-IMPACT targeted sequencing. Regarding the extraction of mutational signatures from targeted sequencing data, Gulhan et al. first proposed the SigMA method (20) in 2019, which is based on likelihood method and machine learning, and is currently the most widely used method. Although the SigProfilerAssignment method (44) proposed by Díaz-Gay et al. can also extract mutational signatures based on targeted mutation data, our analysis revealed that the resulting mutation activity data was relatively sparse and not suitable for subsequent analyses. Therefore, in this study, we ultimately chose the SigMA method for mutational signature extraction.

This study also has several limitations. Firstly, the tumor samples included in this study were derived from several distinct datasets. Although they have all undergone targeted sequencing, biases may arise during the process of data integration and analysis. Secondly, although this study identified several mutational molecular markers related to immunotherapy prognosis, there is a lack of relevant functional experiments to further confirm these findings. Thirdly, the sequencing scope of the targeted sequencing tumor samples included in this study is smaller than that of WES, which may result in the loss of some information. Therefore, prospective WES immunotherapy cohorts and in-depth functional experiments are needed to validate the relevant results of this study.

In summary, this work identified molecular indicators of tumor immunotherapy outcomes at the somatic mutation level and explored their gender differences. These findings may be used to guide the conduct of clinical trials and the development of treatment strategies.

## Data Availability

The original contributions presented in the study are included in the article/**Supplementary Material**. Further inquiries can be directed to the corresponding authors.
