# Supplementary material for: Somatic mutational landscape reveals mutational signatures and significantly mutated genes of cancer immunotherapeutic outcome and sex disparities
Source: Front Immunol. 2024 Nov 1;15:1423796. doi: 10.3389/fimmu.2024.1423796 (PMC11563811; doi:10.3389/fimmu.2024.1423796)
Supplement: Supplementary file 2 [file DataSheet2.docx]

**
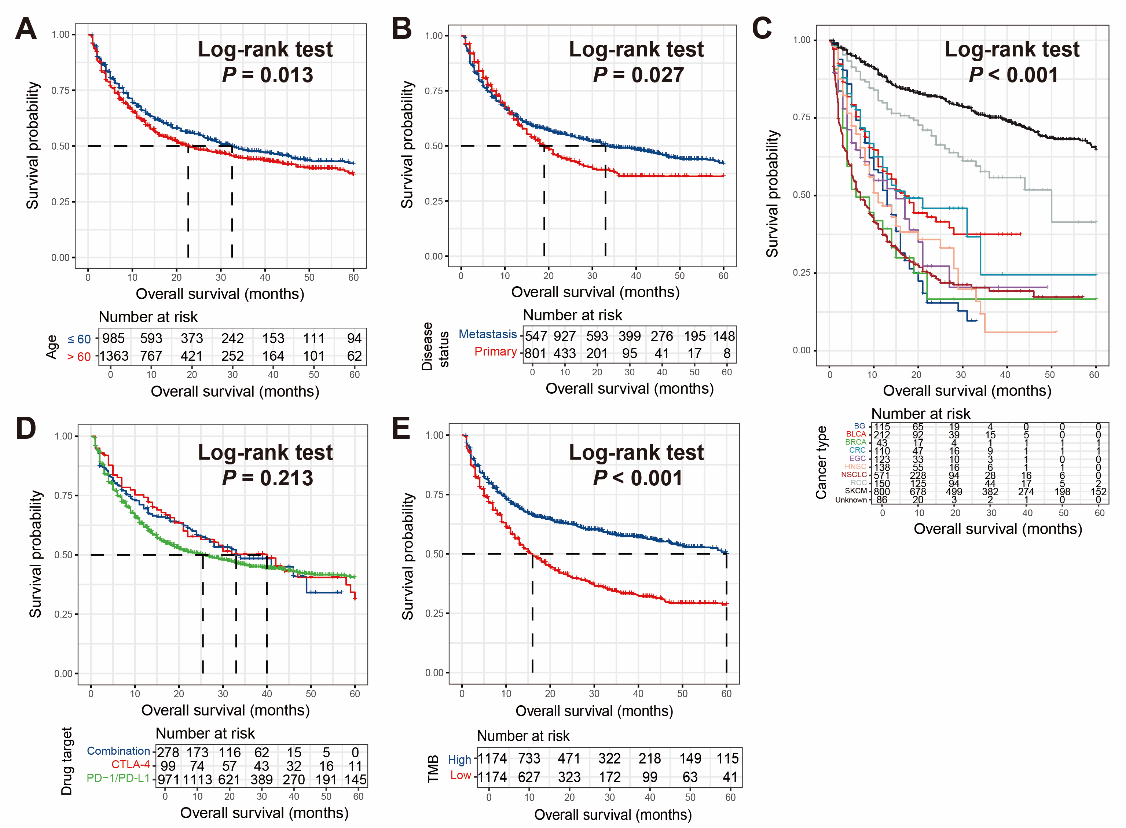
Supplementary Figures**

**
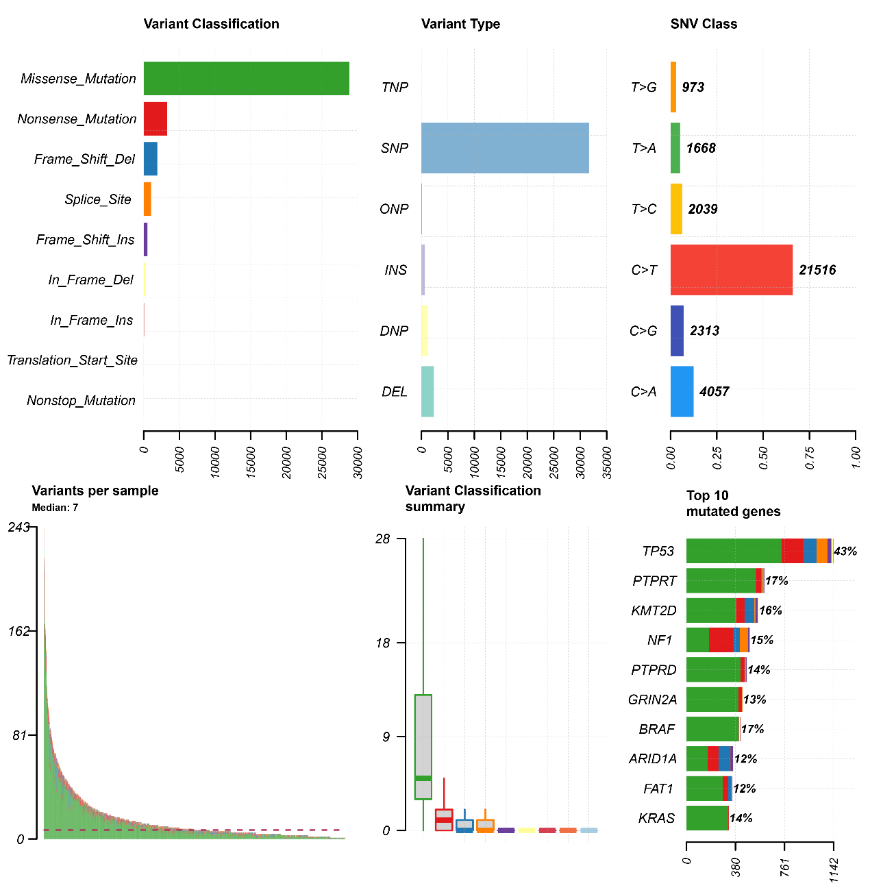
Figure S1. Kaplan-Meier survival analysis of (A) age, (B) disease status, (C) cancer type, (D) drug target, and (E) TMB in the pooled cancer immunotherapy cohort.**

**Figure S2. Detailed mutation classification, mutation type, variants per sample, and frequently mutated genes of integrated cancer immunotherapy cohort.**

**
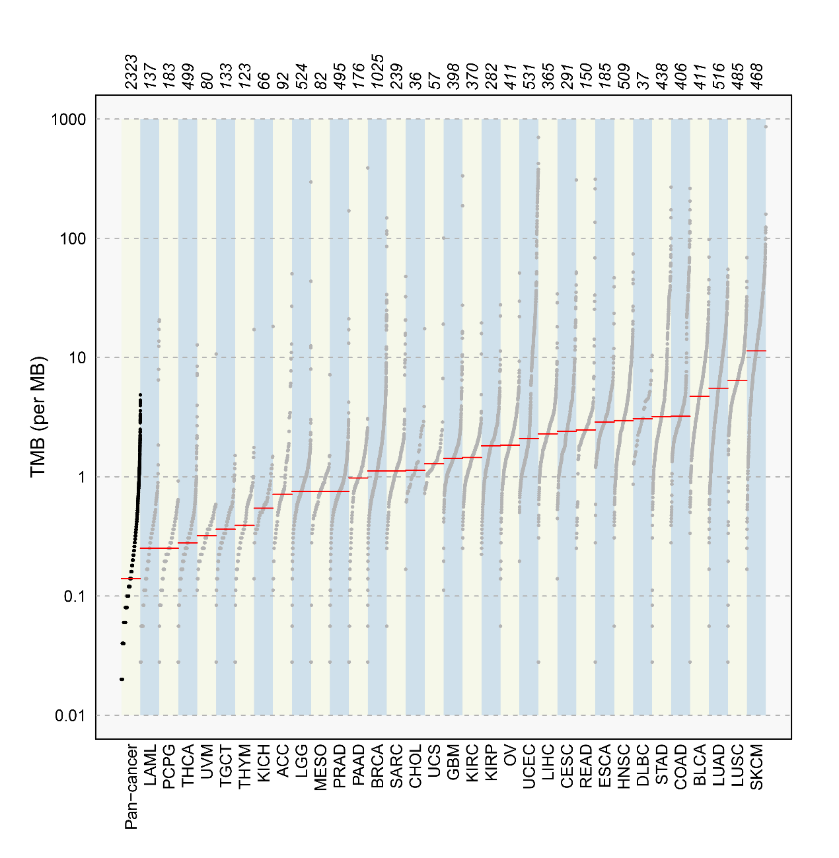

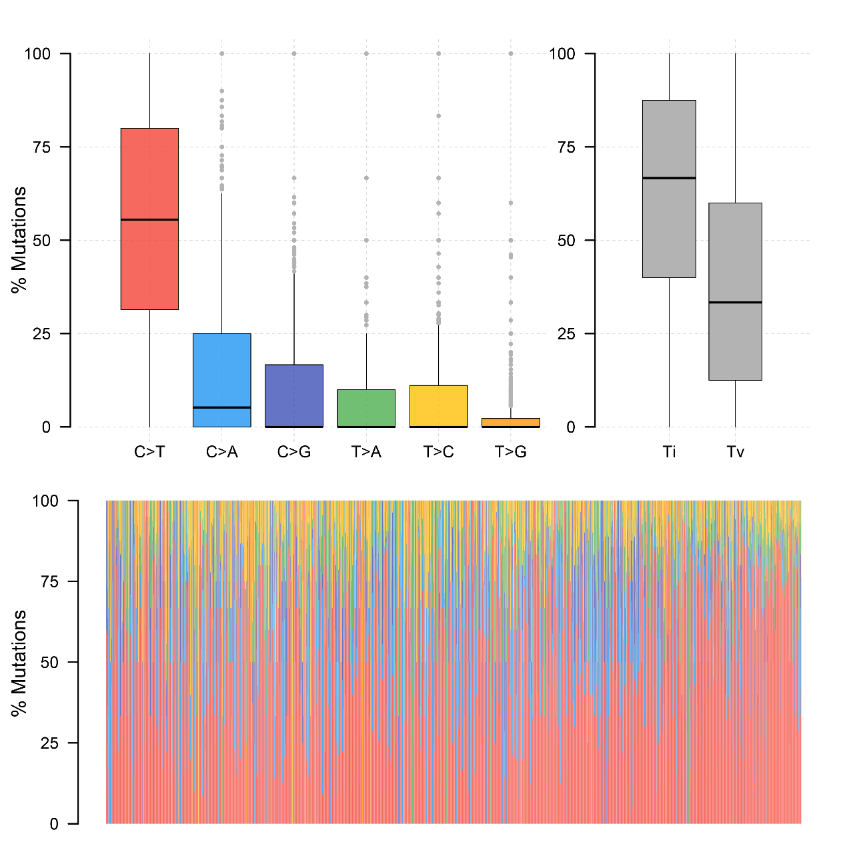
****Figure S3. Detailed SNP transitions and transversions, as well as the types of base substitutions in all samples.**

**Figure S4. Comparison of TMB of the integrated cancer cohort in this study with 33 tumor types in the TCGA database.**

**
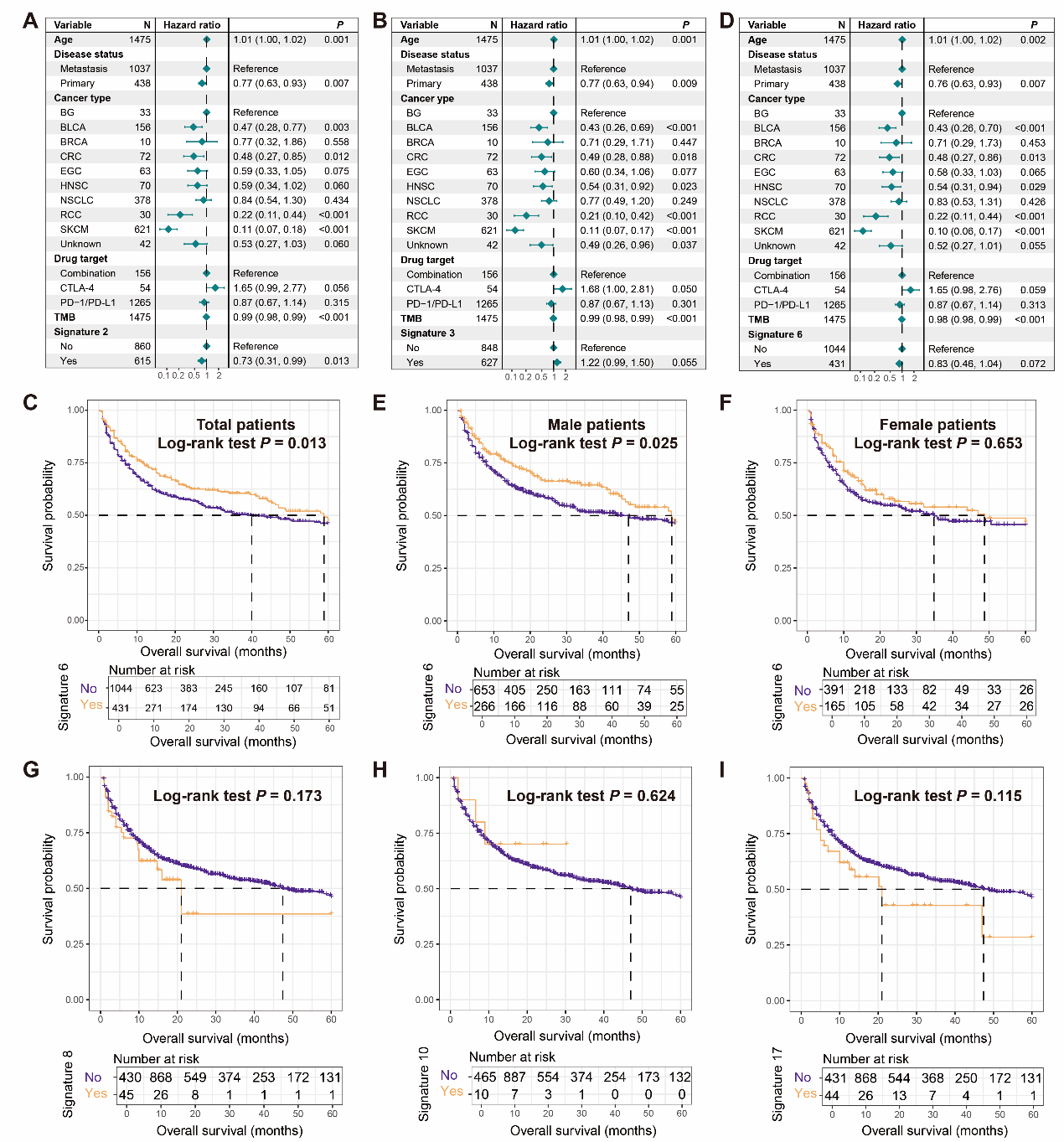
**

**Figure S5. Associations of determined mutational signatures with immunotherapy prognosis.** (A) Multivariate Cox regression model of signature 2. (B) Multivariate Cox regression model of signature 3. (C) Kaplan-Meier survival curves stratified by signature 6 statuses. (D) Multivariate Cox regression model of signature 6. (E) Kaplan-Meier survival curves stratified by signature 6 statuses in male patients. (F) Kaplan-Meier survival curves stratified by signature 6 statuses in female patients. Kaplan-Meier survival curves stratified by (G) signature 8 statuses, (H) signature 10 statuses, and (I) signature 17 statuses.

**
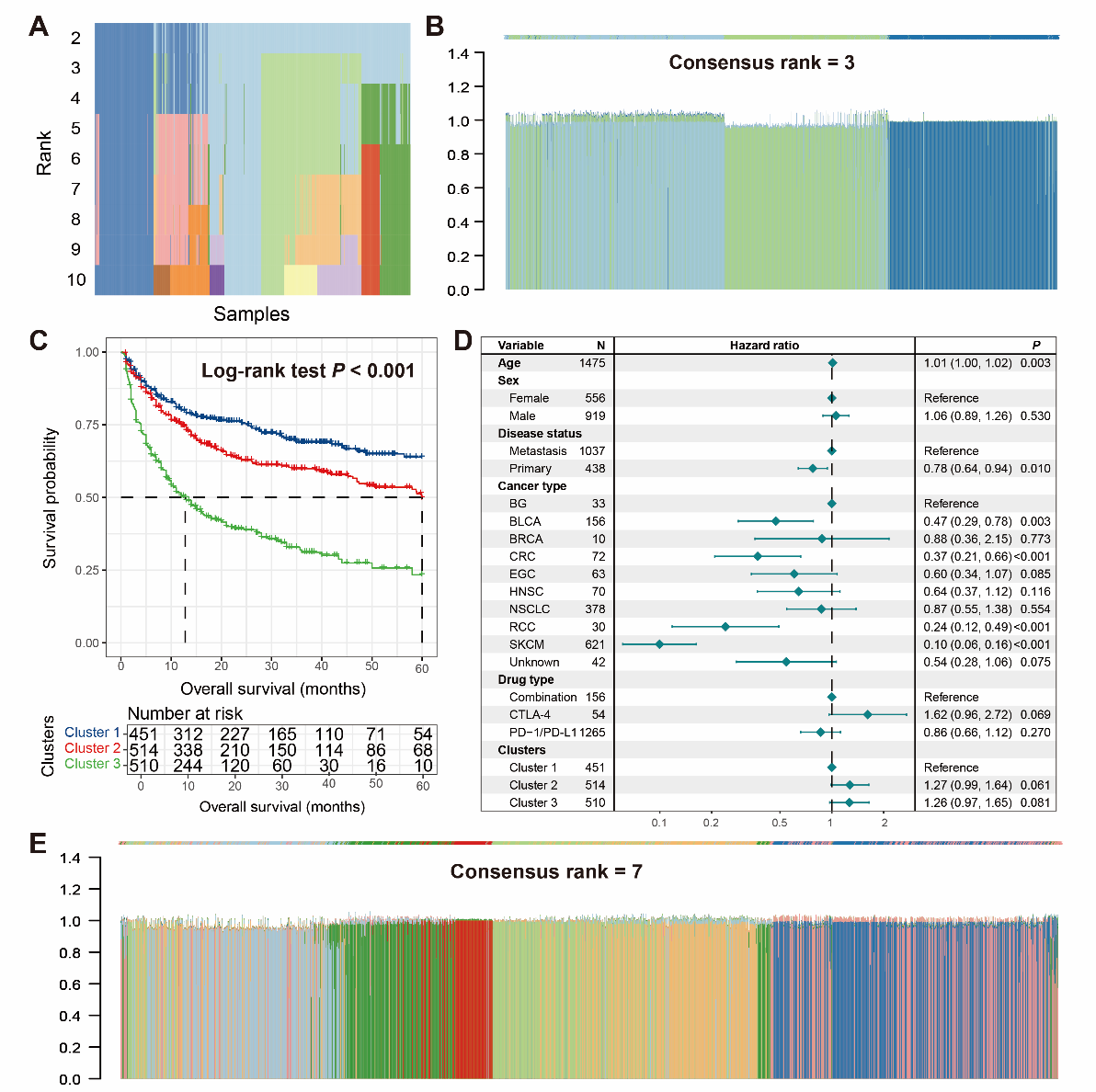
**

**Figure S6. Consensus clustering information and determined molecular subtypes based on mutational signature activities.** (A) Exhibition of clustering tracking plot with cluster numbers selecting from 2 to 10. (B) Detailed clustering consensus between distinct clusters when the number was chosen as 3. (C) Kaplan-Meier survival curves stratified by three molecular clusters. (D) Multivariate Cox regression model of three molecular clusters. (E) Detailed clustering consensus between distinct clusters when the number was chosen as 7.

**
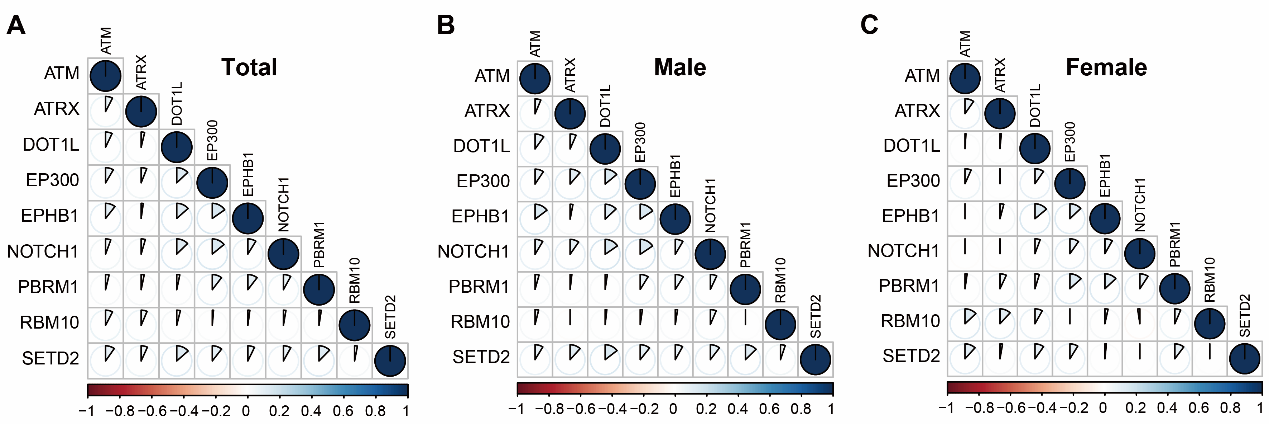

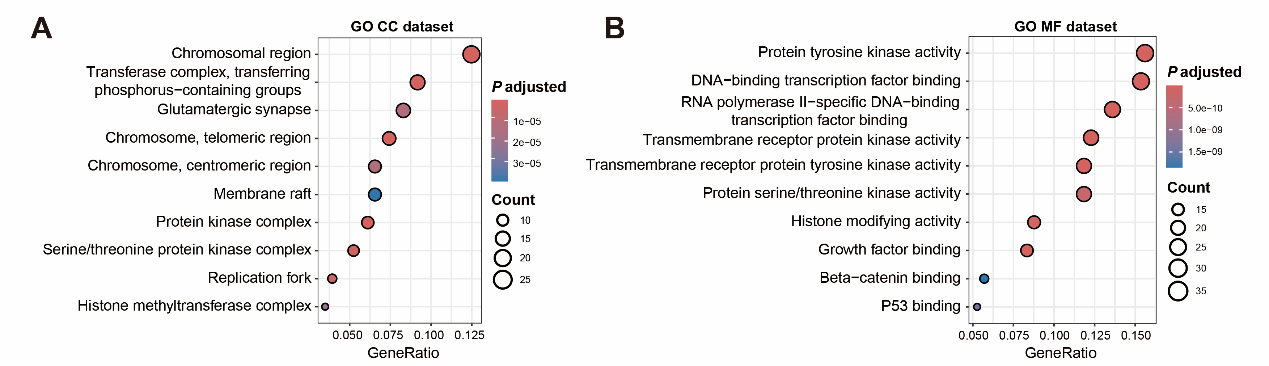
Figure S7. Enrichment analyses of immunotherapeutic prognostic mutated genes base on the circuits from (A) GO CC and (B) GO MF datasets.**

**
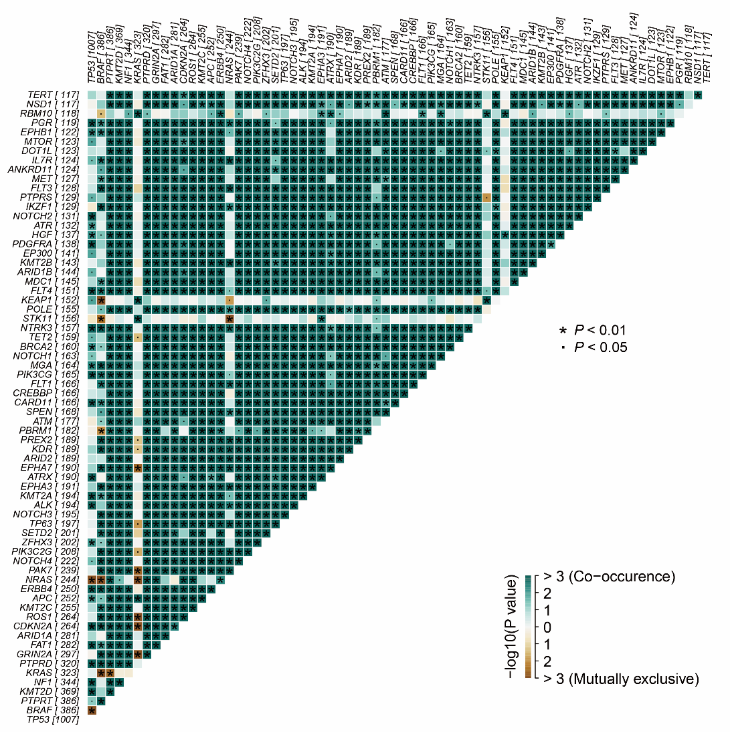
Figure S8. Correlation analyses of determined nine sex-related significantly mutated genes in (A) total cancer patients, (B) male patients, and (C) female patients.**

**Figure S9. Co-occurrence or mutually exclusion of 68 immunotherapeutic prognostic gene mutations in total patients.**

**
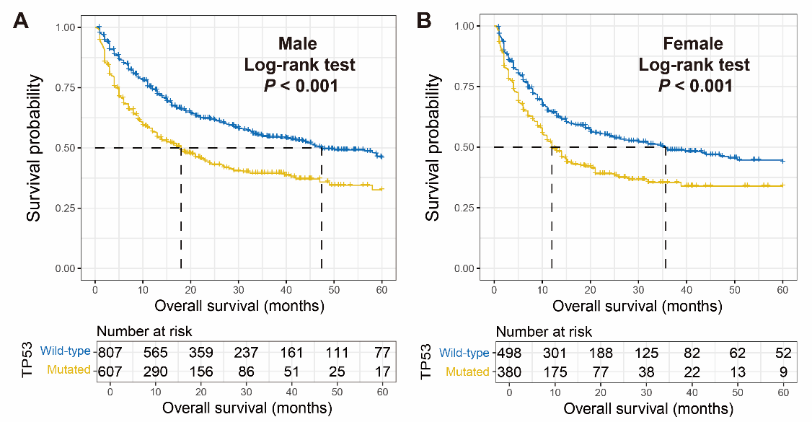
Figure S10. Kaplan-Meier survival curves stratified by TP53 mutation statuses in (A) male patients and (B) female patients.**
